# Comprehensive Evaluation of Serum tRF-17-WS7K092 as a Promising Biomarker for the Diagnosis of Gastric Cancer

**DOI:** 10.1155/2022/8438726

**Published:** 2022-09-19

**Authors:** Xinliang Gu, Yu Zhang, Yuejiao Huang, Shaoqing Ju

**Affiliations:** ^1^Medical School of Nantong University, Nantong University, Nantong, China; ^2^Department of Laboratory Medicine, Affiliated Hospital of Nantong University, Nantong, China; ^3^Research Center of Clinical Medicine, Affiliated Hospital of Nantong University, Nantong, China; ^4^Department of Medical Oncology, Affiliated Hospital of Nantong University, Nantong, China

## Abstract

**Background:**

Gastric cancer (GC) is a malignant tumor of the gastrointestinal system. Since the early symptoms of GC are not obvious and lack efficient diagnostic markers, it is urgent to find new diagnostic markers with good sensitivity and specificity. tRNA-derived small RNAs (tsRNAs) are an emerging class of small noncoding RNAs with good abundance in body fluids. We aim to find new tsRNAs as biomarkers for GC diagnosis.

**Methods:**

High-throughput sequencing was used to identify differentially expressed tsRNAs in GC tissues, and quantitative real-time PCR was used to detect the expression level of tRF-17-WS7K092. Agarose gel electrophoresis and Sanger sequencing were performed to verify the characteristics of tRF-17-WS7K092. The diagnostic efficacy of tRF-17-WS7K092 was analyzed by the receiver operating characteristic curve.

**Results:**

In this study, the expression levels of tRF-17-WS7K092 were significantly increased in GC tissues, cells, and serum. After GC surgery, the expression level of serum tRF-17-WS7K092 decreased, and its high expression was associated with low survival rates. In addition, the expression level of serum tRF-17-WS7K092 was correlated with the T stage, TNM stage, lymph node metastasis, and nerve/vascular invasion and could distinguish GC patients from gastritis patients and healthy donors as well.

**Conclusions:**

The expression of serum tRF-17-WS7K092 was significantly increased in GC and decreased after GC surgery, suggesting that serum tRF-17-WS7K092 may serve as a promising biomarker for the diagnostic and prognostic monitoring of GC.

## 1. Background

Gastric cancer (GC) is a malignant tumor of the gastrointestinal system that originates from the epithelium of the gastric mucosa, with the fourth- and fifth-highest incidence and mortality rates of malignant tumors, respectively [1]. The early symptoms of GC are similar to those of gastritis with no obvious symptoms and are often neglected [2]. The traditional tumor markers such as carcinoembryonic antigen (CEA), carbohydrate antigen 199 (CA199), and carbohydrate antigen 724 (CA724) are commonly used to detect GC, but their sensitivity (SEN) and specificity (SPE) are not enough [3]. Therefore, it is urgent to search for new biomarkers with good SEN and SPE for the clinical diagnosis of GC.

Transfer RNA-derived small RNAs (tsRNAs) are a class of small noncoding RNAs produced by specific ribonucleic acid endonucleases cleaved at different sites on precursor transfer RNAs (tRNAs) or mature tRNAs and can be divided into two categories depending on the enzymatic site: tRNA-derived fragments (tRFs) and tRNA halves (tiRNAs) [4]. tsRNAs can play biological roles through various regulatory mechanisms, including interaction with proteins or mRNAs to regulate the gene expression, epigenetic modifications, and cell cycle [4]. With the development of high-throughput sequencing technology, more and more studies have found that the dysregulation of tsRNAs is closely related to tumor development. Consequently, it is essential to find the dysregulated tsRNAs in GC and explore their specific mechanisms to diagnose GC. For instance, tRF-33-P4R8YP9LON4VDP was found to be significantly downregulated in GC tissues and plasma. Subsequently, cell function experiments revealed that this molecule can inhibit GC cell proliferation and migration, promote GC cell apoptosis, and alter the cell cycle [5]. Zhu et al. found that tRF-18-79MP9P04 is a promising diagnostic marker for GC and can inhibit the proliferation, migration, and cell cycle of GC cells by regulating the PTEN/PI3K/AKT signaling pathway [6]. Thus, we have a strong interest in whether tsRNAs could serve as a novel biomarker for the diagnostic and prognostic monitoring of GC.

In this study, we conducted high-throughput sequencing on three pairs of GC tissues and adjacent nontumor tissues to identify dysregulated tsRNAs and chose highly expressed tRF-17-WS7K092 for further research. We found that the expression level of tRF-17-WS7K092 was increased significantly in the serum of GC patients and could distinguish between GC patients and gastritis patients or healthy donors. Furthermore, the tRF-17-WS7K092 expression was decreased after GC surgery, indicating that tRF-17-WS7K092 could dynamically monitor GC patients. Meanwhile, receiver operating characteristic (ROC) curve analysis showed that serum tRF-17-WS7K092 has good diagnostic efficacy, which kept increasing after combining with other tumor markers. Therefore, tRF-17-WS7K092 has the potential to become a biomarker for the diagnostic and prognostic monitoring of GC.

## 2. Methods

### 2.1. High-Throughput Sequencing

Total RNA was isolated using TRIzol. Small RNA libraries were constructed using the NEBNext® Multiplex Small RNA Library Prep Set for Illumina. Libraries were sequenced using the HiSeq 2500 SE50 mode.

### 2.2. Clinical Specimens

All of the serum samples, including 136 GC patients, 40 gastritis patients, 136 healthy donors, and 39 GC patients after surgery, were collected from the Department of Laboratory Medicine, Affiliated Hospital of Nantong University, from 2016 to 2020. All patients in this study were clinically diagnosed with GC or gastritis and were not treated with any preoperative therapy. 20 pairs of GC tissues and adjacent nontumor tissues were collected from the Department of Pathology, Affiliated Hospital of Nantong University, and all tissues were diagnosed as GC by pathologists. All of the tissues were immediately frozen in liquid nitrogen after resection and then transferred to a -80°C refrigerator for storage. The ethics committee of the local hospital (ethical review report number: 2018-L055) approved this study. Informed consent was obtained from all participants prior to the clinical trial, and permission was given for publication.

### 2.3. Cell Culture

Human gastric epithelial cells (GES-1) and three GC cell lines (MKN-45, AGS, and MKN-1) were obtained from the Chinese Academy of Sciences (Shanghai, China) and were cultured in the RPMI-1640 medium (Corning, USA) with 10% fetal bovine serum (Gibco, USA) in an incubator at 37°C with 5% CO_2_.

### 2.4. Total RNA Extraction, cDNA Synthesis, and Quantitative Real-Time PCR

Serum total RNA was extracted using a rapid blood total RNA extraction kit (BioTeke Corporation, China), while total RNA from tissues and cells was extracted using a TRIzol reagent (Invitrogen, Germany). cDNA was produced using the RevertAid RT Reverse Transcription Kit (Thermo Fisher Scientific, USA), and quantitative real-time PCR (qRT-PCR) was performed on ABI QuantStudio 5 with the 20 *μ*L reaction system, which included 10 *μ*L ChamQ Universal SYBR qPCR Master Mix (Vazyme Biotech Co., Ltd., China), 1 *μ*L primers, 3 *μ*L enzyme-free water, and 5 *μ*L cDNA. U6 was used as an internal reference, and the expression of tRF-17-WS7K092 was calculated by the 2^−∆∆CT^ method. All primers used in the study were synthesized by RiboBio (Guangzhou, China).

### 2.5. Statistical Analysis

All of the statistical analyses in this study were performed using SPSS Statistics Version 20.0 (IBM SPSS Statistics, Chicago, USA) and GraphPad Prism v8.0 (GraphPad Software, San Diego, CA). tRF-17-WS7K092 expression in different groups was expressed as mean ± standard deviation (SD), and all of the data in this study were first tested for normality. Two independent groups were compared using the Mann-Whitney *U* test, and the expression level of tRF-17-WS7K092 in GC cells was compared using one-way analysis of variance (ANOVA). The Kruskal-Wallis *H* test was used to analyze the expression level of tRF-17-WS7K092 in GC patients, gastritis patients, and healthy donors. The Wilcoxon matched-pairs signed rank test was used for analyzing the expression level of tRF-17-WS7K092 in preoperative patients and postoperative patients, as well as GC tissues and adjacent tissues. The log-rank test was used to assess the significance of the survival data between different groups. The Youden index was used to define the cutoff value, and the area under the ROC curve (AUC) was used to evaluate the diagnostic performance. Differences were considered statistically significant when *P* < 0.05 [7].

## 3. Results

### 3.1. The Expression Profile of tsRNAs in GC Tissues

**Figure 1:**
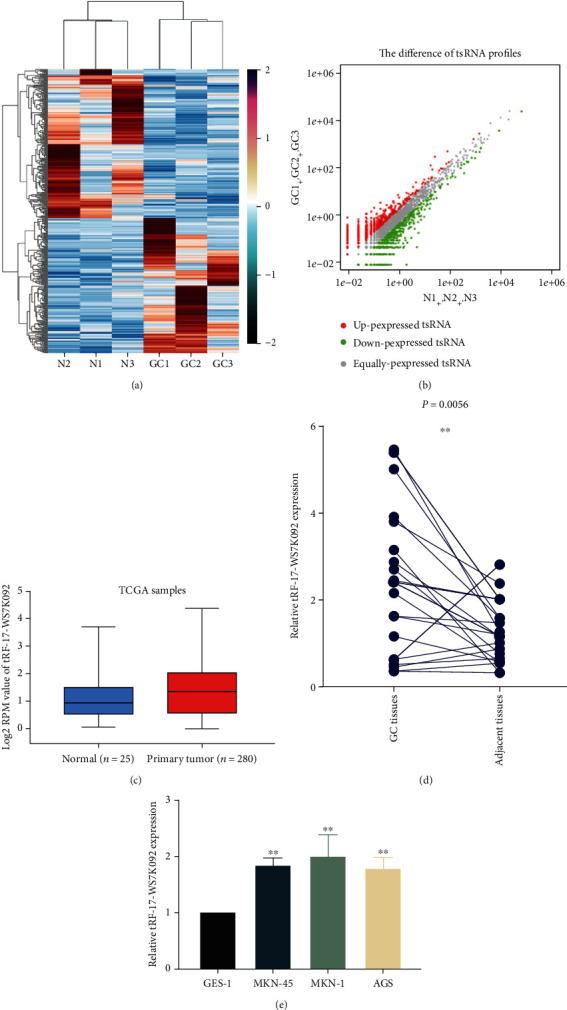
The expression profile of tsRNAs in GC tissues. (a) Heat map of differential tsRNAs in three pairs of GC tissues and adjacent nontumor tissues analyzed by tsRNA sequencing. (b) Differential expression map of tsRNAs in three pairs of GC tissues and adjacent nontumor tissues analyzed by tsRNA sequencing. (c) Online validation of tRF-17-WS7K092 in TCGA stomach adenocarcinoma database. (d) Expression level of tRF-17-WS7K092 in twenty pairs of GC tissues. (e) Expression level of tRF-17-WS7K092 in GC cells. ^∗∗^*P* < 0.01.

To identify the dysregulated tsRNAs in GC tissues, we conducted high-throughput sequencing of tsRNAs in three pairs of GC tissues and adjacent nontumor tissues. Through the heat map and differential expression map, the sequencing results were visualized and we chose highly expressed tRF-17-WS7K092 for further research (Figures 1(a) and 1(b)). We performed online validation of tRF-17-WS7K092 in TCGA stomach adenocarcinoma database and found that the expression trend was consistent with the sequencing results (Figure 1(c)). Subsequently, the qRT-PCR detection revealed that tRF-17-WS7K092 expression was significantly increased in twenty pairs of GC tissues (*P* = 0.0056), consistent with the sequencing results also (Figure 1(d)). Meanwhile, the expression of tRF-17-WS7K092 increased in GC cells compared with GES-1 (Figure 1(e)).

### 3.2. tRF-17-WS7K092 Is a Kind of 3′-tRF

Using the UCSC Genome Browser database, we found that tRF-17-WS7K092 was located at chr17 (q21.32), with 47,269,890-47,269,961 (Additional file 1: Figure S1). MINTbase v2.0 (https://cm.jefferson.edu/MINTbase/) showed that tRF-17-WS7K092 was a 3′-tRF with the length of 17 nt (5′-TCTCGGTGGGACCTCCA-3′), which was derived from tRNA-Gln-TTG (Additional file 1: Figure S1B), and the cleavage site was on the T-loop (Additional file 1: Figure S1C). Then, agarose gel electrophoresis (AGE) showed a single electrophoretic band of about 80 bp for the qRT-PCR product, and we performed Sanger sequencing on it, revealing that the product contained the complete sequence of tRF-17-WS7K092 consistent with that in MINTbase (Additional file 1: Figures S1D and S1E).

### 3.3. Comprehensive Evaluation of the Detection Method of tRF-17-WS7K092

To investigate whether the detection method of tRF-17-WS7K092 could be applied to clinical practice, we conducted a comprehensive evaluation. First, we repeatedly freeze-thawed the mixed serum specimens 0, 1, 3, 5, and 10 times and left them at room temperature (about 25°C) for 0, 6, 8, 12, and 24 hours; the result showed that the expression level of tRF-17-WS7K092 has no significant change, indicating the detection method of tRF-17-WS7K092 has good stability and repeatability and is not easily affected by these factors (Additional file 1: Figures S2A and S2B). Additionally, to detect the linear range of tRF-17-WS7K092 and U6, we diluted the cDNA of GC patients for qRT-PCR. The results showed that the standard curves for tRF-17-WS7K092 and U6 were *y* = −1.549*x* + 28.43, *R*^2^ = 0.9906 and *y* = −3.083*x* + 20.17, *R*^2^ = 0.9907, respectively, demonstrating good linearity of the detection method (Additional file 1: Figures S2C and S2D). Then, we used the mixed serum to assess the precision of tRF-17-WS7K092, finding that the intra-assay coefficient of variation (CV) and interassay CV are 1.8% and 2.19%, respectively (Additional file 1: Table S1). Additionally, the smooth amplification curve and single peak of the melting curve further confirmed the accuracy and specificity of the method (Additional file 1: Figures S2E and S2F). All the above experiments suggested that the detection method of tRF-17-WS7K092 has good stability and specificity, which can be used for clinical practice.

### 3.4. Potential of Serum tRF-17-WS7K092 as a Diagnostic and Prognostic Marker and the Correlation with Clinicopathological Parameters

**Figure 2:**
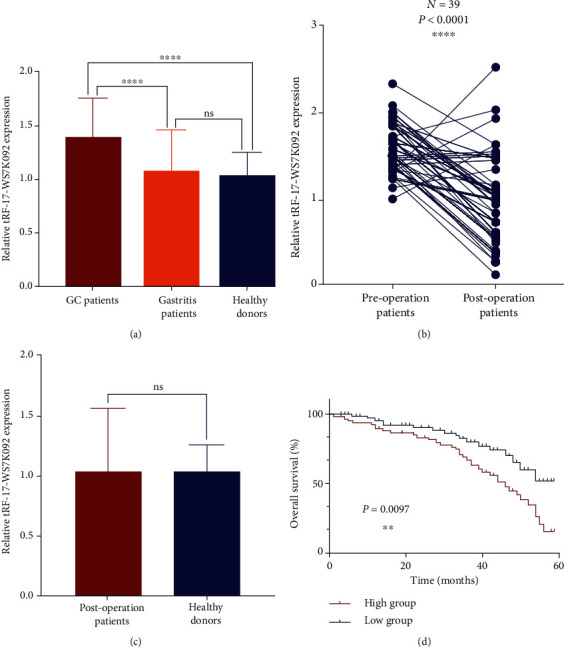
Potential of serum tRF-17-WS7K092 as a diagnostic and prognostic marker. (a) The expression level of serum tRF-17-WS7K092 in GC patients (*N* = 136), gastritis patients (*N* = 40), and healthy donors (*N* = 136). (b) Comparison of the serum tRF-17-WS7K092 expression level in GC patients before and after surgery. (c) Comparison of the serum tRF-17-WS7K092 expression level between postoperative GC patients and healthy donors. (d) Kaplan-Meier method verifies the prognostic value of tRF-17-WS7K092. ^ns^*P* > 0.05, ^∗∗^*P* < 0.01, and ^∗∗∗∗^*P* < 0.0001.

To explore the diagnostic potential of tRF-17-WS7K092, we collected serum samples from 136 GC patients, 40 gastritis patients, and 136 healthy donors. The results of qRT-PCR showed that the expression level of tRF-17-WS7K092 in GC patients was significantly increased compared with that in healthy donors and gastritis patients. In contrast, the expression level showed no significant difference between gastritis patients and healthy donors (Figure 2(a)). Then, we divided the 136 GC patients into two groups according to the median expression of tRF-17-WS7K092: relative high group (expression > 1.390, *n* = 68) and relative low group (expression < 1.390, *n* = 68). The chi-squared test was used to analyze the correlation between the expression level of tRF-17-WS7K092 and the clinicopathological parameters. As shown, the tRF-17-WS7K092 expression was positively correlated with the T stage, lymph node status, TNM stage, and nerve/vascular invasion but led to no significant relationship with sex, age, tumor size, and differentiation grade (Table 1). To investigate the prognostic monitoring value of tRF-17-WS7K092 in GC, we collected 39 serum samples from GC patients after surgery and compared the tRF-17-WS7K092 expression with that of the corresponding preoperative patients. It was found that the tRF-17-WS7K092 expression was significantly decreased after GC surgery and had no significant difference with healthy donors (Figures 2(b) and 2(c)). Besides, the survival curve showed that the low group was correlated with a high survival rate (Figure 2(d)).

### 3.5. Evaluation of the Diagnostic Efficacy of Serum tRF-17-WS7K092 in GC

**Figure 3:**
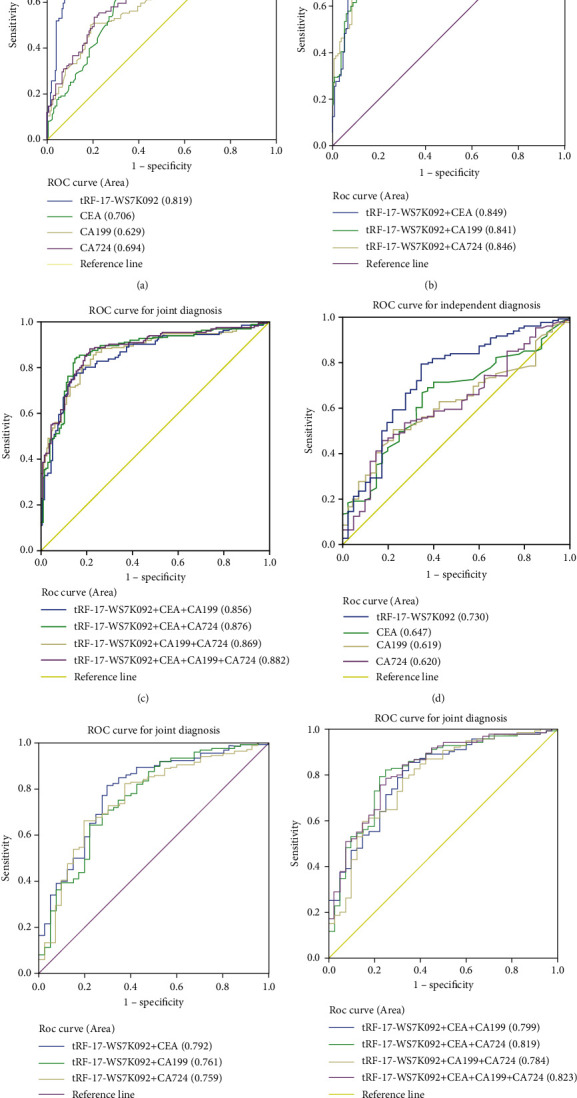
Evaluation of the diagnostic efficacy of serum tRF-17-WS7K092 in GC. (a) ROC analysis of tRF-17-WS7K092, CEA, CA199, and CA724 in the independent diagnosis of GC patients and healthy donors. (b, c) ROC analysis of tRF-17-WS7K092, CEA, CA199, and CA724 in the joint diagnosis of GC patients and healthy donors. (d) ROC analysis of tRF-17-WS7K092, CEA, CA199, and CA724 in the independent diagnosis of GC patients and gastritis patients. (e, f) ROC analysis of tRF-17-WS7K092, CEA, CA199, and CA724 in the joint diagnosis of GC patients and gastritis patients.

To evaluate the diagnostic efficacy of serum tRF-17-WS7K092 in GC, we first performed ROC analysis of serum tRF-17-WS7K092 and traditional tumor markers (CEA, CA199, and CA724) in 136 GC patients and 136 healthy donors. Results showed that the AUC of tRF-17-WS7K092 was 0.819 (95% confidence interval (CI): 0.766-0.873), higher than that of CEA (0.706, 95% CI: 0.644-0.767), CA199 (0.629, 95% CI: 0.562-0.696), and CA724 (0.694, 95% CI: 0.632-0.757) (Figure 3(a)). Meanwhile, the SEN and SPE of tRF-17-WS7K092 were 0.77 and 0.84, while those of CEA, CA199, and CA724 were 0.60 and 0.71, 0.51 and 0.80, and 0.56 and 0.74 (Table 2). Then, we combined tRF-17-WS7K092 with CEA, CA199, and CA724, finding that the AUC and SEN increased when tRF-17-WS7K092 was combined with other tumor markers (Figure 3(b)). When three markers were combined, the diagnostic efficacy improved further, and the AUC reached the highest (0.882) when the four were combined (Figure 3(c)). The results above suggested that tRF-17-WS7K092 has good diagnostic efficacy in distinguishing GC patients from healthy donors and can be further improved when joint.

The early symptoms of GC are similar to those of gastritis, so whether serum tRF-17-WS7K092 could identify GC patients from gastritis patients is significant. Then, we performed ROC analysis on serum tRF-17-WS7K092 and the traditional tumor markers in 136 GC patients and 40 gastritis patients and came to similar conclusions. The AUC of tRF-17-WS7K092 was 0.730 (95% CI: 0.637-0.823), higher than that of CEA (0.647, 95% CI: 0.555-0.739), CA199 (0.619, 95% CI: 0.530-0.708), and CA724 (0.620, 95% CI: 0. 526-0.715) (Figure 3(d)). The SEN of tRF-17-WS7K092 was 0.79, higher than that of other tumor markers (Additional file 1: Table S2). When we combined tRF-17-WS7K092 with other markers, the diagnostic efficacy increased constantly, and the AUC reached the highest of 0.823 when combined with CEA, CA199, and CA724 (Figures 3(e) and 3(f)). The above research indicates that serum tRF-17-WS7K092 could distinguish between GC patients and gastritis patients, and the diagnostic value keeps increasing when combined with other tumor markers.

### 3.6. Prediction of the Downstream Regulation Mechanism of tRF-17-WS7K092

**Figure 4:**
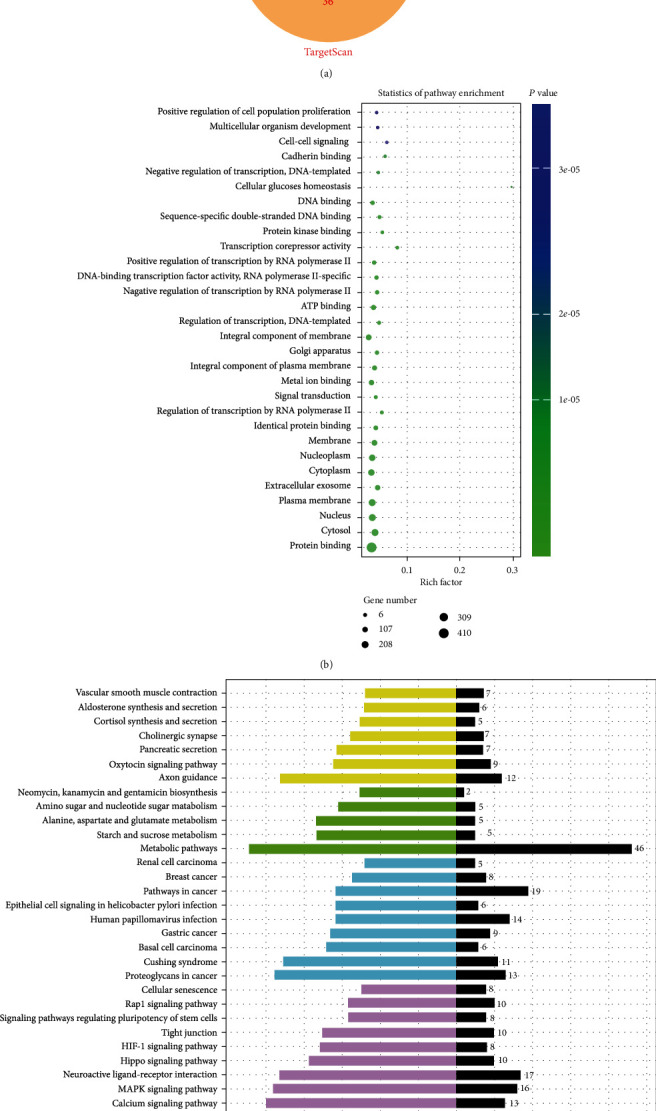
Prediction of the downstream regulation mechanism of tRF-17-WS7K092. (a) The potential target genes of tRF-17-WS7K092. (b) Functional enrichment analysis of GO of the potential target genes. (c) Enrichment analysis of the KEGG biological pathway of the potential target genes.

To investigate the potential mechanism of tRF-17-WS7K092 in GC, we used miRanda, TargetScan, and RNAhybrid databases to find the potential target genes of tRF-17-WS7K092. As shown in Figure 4(a), the 133 genes at the intersection of the three databases were most likely to be the target genes of tRF-17-WS7K092. Besides, functional enrichment analysis of Gene Ontology (GO) revealed that the potential target genes might have functions in protein binding, transcription corepressor activity, and cellular glucose homeostasis (Figure 4(b)). Enrichment analysis of the Kyoto Encyclopedia of Genes and Genomes (KEGG) biological pathway suggested that the axon guidance, metabolic pathways, and calcium signaling pathway were enriched significantly (Figure 4(c)). Still, the fundamental regulatory mechanism of tRF-17-WS7K092 remains to be investigated in the future.

## 4. Discussion

GC is a severe malignancy worldwide and added more than one million new cases in just one year. Furthermore, it is the most common cancer among men in Southern Central Asian countries and the leading cause of cancer-related deaths [8]. Chronic infection with H. pylori is the main cause of GC [9]; with improved living and hygiene conditions and the widespread use of antimicrobials, the prevalence of H. pylori has declined over the past few decades, as has the incidence and mortality of GC [10]. As a convenient technique for tumor sampling, liquid biopsy has gradually replaced invasive methods for diagnosing and detecting cancer in the past decade, which opens up new avenues in the detection and continuous monitoring of cancer patients [11]. However, due to the lack of clinical symptoms and screening markers and the late diagnosis of GC, most patients were already in an advanced stage when diagnosed, and the 5-year survival rate was low [12, 13]. Hence, it is essential to find promising biomarkers with great SEN and SPE to diagnose GC in the early stage.

In recent years, with the development of high-throughput sequencing technology, more and more noncoding RNAs have been discovered [14]. Some of them were found to act as markers for tumor diagnosis. For instance, hsa_circ_000780 was downregulated significantly in GC tissues and gastric fluid specimens. It might become a biomarker of GC screening [15]. Besides, a lncRNA called NR038975 was upregulated in the serum of GC patients and involved in GC progression, which might be a potential therapeutic target and a new diagnostic marker for GC [16]. However, these noncoding RNAs are still of low abundance in the body fluids of GC patients and are not sufficient for clinical diagnosis. Therefore, it is urgent to find novel biomarkers of high abundance in the body fluids of GC patients.

tsRNAs were first discovered in the late 1970s. At that time, tsRNAs were considered random degradation products and did not attract widespread attention [14, 17, 18]. Recently, numerous experiments and studies have demonstrated that tsRNAs are actually derived fragments of pre-tRNAs or mature tRNAs produced by specific cleavage under specific environments and have various biological functions [19]. Moreover, many research studies revealed that tsRNAs are characterized by high abundance and stability in the body fluids of cancer patients and have great potential as biomarkers for cancer diagnosis and prognosis as well as therapeutic targets [20–22]. To search for novel GC biomarkers, we conducted high-throughput sequencing on three paired GC tissues, and the highly expressed tRF-17-WS7K092 was chosen for further research. Subsequently, we detected the expression of serum tRF-17-WS7K092 and constructed ROC curves, which found that the expression of tRF-17-WS7K092 was significantly increased in GC serum and could distinguish between GC patients and gastritis patients or healthy donors. After combining with other traditional tumor markers, the diagnostic efficacy of tRF-17-WS7K092 was further improved. In addition, the expression of tRF-17-WS7K092 was decreased after GC surgery, and the high expression level of tRF-17-WS7K092 was related to a low survival rate, indicating its potential for dynamic monitoring for the prognosis of GC patients. The above experiments suggest that serum tRF-17-WS7K092 can distinguish between GC patients and gastritis patients or healthy donors and serve not only as a diagnostic marker but also as a prognostic marker for GC.

In this study, we found that tRF-17-WS7K092, which was highly expressed in GC tissues using high-throughput sequencing technology, was also elevated in GC serum and has the potential to become a diagnostic and prognostic biomarker for GC. However, we only found that tRF-17-WS7K092 was aberrantly highly expressed in GC, but its function and the mechanism that affects GC progression remain unclear. Mo et al. found that after downregulation of 5′-tiRNA^Val^ in breast cancer (BC), further studies showed that it was associated with the TNM stage and lymph node metastasis and could act as a tumor suppressor to inhibit the proliferation and invasion of BC cells [23]. Therefore, we similarly analyzed the correlation between the expression of tRF-17-WS7K092 and the clinicopathological parameters and found that it was positively correlated with the T stage, TNM stage, lymph node metastasis, and nerve/vascular invasion, suggesting that tRF-17-WS7K092 may have the ability to promote the proliferation and metastasis of GC cells, which requires us to conduct subsequent studies on it. Notably, regulation of gene expression is one of the most important functions of tsRNAs. tsRNAs could be involved in the formation of RNA-induced silencing complexes and regulate mRNA stability through posttranscriptional pathways by binding to the 3′ untranslated region of target genes [24]. For instance, tRF-3017A binds to the AGO protein to regulate the oncogene NELL2 expression and promote the migration and invasion of GC cells [25]. So, we used three databases to predict the potential targeted genes of tRF-17-WS7K092, and KEGG and GO databases were used to predict the pathways and functions of these target genes enriched, contributing to future studies on the specific molecular mechanisms of tRF-17-WS7K092 in GC progression.

In conclusion, we found that tRF-17-WS7K092 has the potential to become a novel biomarker for the diagnostic and prognostic monitoring of GC, but its specific function and mechanism in GC remain to be further investigated.

## 5. Conclusions

In summary, our study revealed for the first time that the expression level of tRF-17-WS7K092 was significantly upregulated in GC tissues, cells, and serum. Additionally, serum tRF-17-WS7K092 can distinguish GC patients from gastritis patients and healthy donors, and its expression level decreased after GC surgery, which can be used for dynamic monitoring of GC patients. Our research suggested that serum tRF-17-WS7K092 has the potential to become a promising biomarker for the diagnostic and prognostic monitoring of GC, but its specific function and mechanism remain to be further researched.

## Figures and Tables

**Table 1:** Clinicopathological analysis of tRF-17-WS7K092.

Parameter		No. of patients	tRF-17-WS7K092 (high)	tRF-17-WS7K092 (low)	*P* value
Sex	Male	99	52	47	0.335
	Female	37	16	21	

Age (year)	<60	36	17	19	0.697
	≥60	100	51	49	

Tumor size	<5	98	44	54	0.056
	≥5	38	24	14	

Differentiation grade	Well-moderate	63	27	36	0.122
	Poor-undifferentiation	73	41	32	

T stage	T1-T2	71	27	44	0.004^∗∗^
	T3-T4	65	41	24	

Lymph node status	Positive	85	52	33	0.001^∗∗^
	Negative	51	16	35	

TNM stage	I-II	76	28	48	0.001^∗∗^
	III-IV	60	40	20	

Nerve/vascular invasion	Positive	84	50	34	0.005^∗∗^
	Negative	52	18	34	

^∗∗^ *P* < 0.01.

**Table 2:** The diagnostic performance of tRF-17-WS7K092, CEA, CA199, and CA724 in differentiating GC patients from healthy donors.

	SEN	SPE	ACCU	PPV	NPV
tRF-17-WS7K092	0.77 (105/136)	0.84 (114/136)	0.81 (219/272)	0.83 (105/127)	0.79 (114/145)
CEA	0.60 (81/136)	0.71 (96/136)	0.65 (177/272)	0.67 (81/121)	0.64 (96/151)
CA199	0.51 (69/136)	0.80 (109/136)	0.65 (178/272)	0.72 (69/96)	0.62 (109/176)
CA724	0.56 (76/136)	0.74 (101/136)	0.65 (177/272)	0.68 (76/111)	0.63 (101/161)
tRF-17-WS7K092+CEA	0.90 (122/136)	0.60 (81/136)	0.75 (203/272)	0.69 (122/177)	0.85 (81/95)
tRF-17-WS7K092+CA199	0.91 (124/136)	0.68 (92/136)	0.79 (216/272)	0.74 (124/168)	0.88 (92/104)
tRF-17-WS7K092+CA724	0.90 (123/136)	0.63 (86/136)	0.77 (209/272)	0.71 (123/173)	0.87 (86/99)
tRF-17-WS7K092+CEA+CA199	0.95 (129/136)	0.49 (67/136)	0.72 (196/272)	0.65 (129/198)	0.91 (67/74)
tRF-17-WS7K092+CEA+CA724	0.96 (131/136)	0.44 (60/136)	0.70 (191/272)	0.63 (131/207)	0.92 (60/65)
tRF-17-WS7K092+CEA+CA199+CA724	0.98 (133/136)	0.38 (51/136)	0.68 (184/272)	0.61 (133/218)	0.94 (51/54)

SEN: sensitivity; SPE: specificity; ACCU: overall accuracy; PPV: positive predictive value; NPV: negative predictive value.

## Data Availability

Data are available upon reasonable request. The data used in the current study are available from the corresponding author upon reasonable request.

## Abbreviations

GC: Gastric cancer
CEA: Carcinoembryonic antigen
CA199: Carbohydrate antigen 199
CA724: Carbohydrate antigen 724
SEN: Sensitivity
SPE: Specificity
tsRNAs: Transfer RNA-derived small RNAs
tRNAs: Transfer RNAs
tRFs: tRNA-derived fragments
tiRNAs: tRNA halves
ROC: Receiver operating characteristic
qRT-PCR: Quantitative real-time PCR
SD: Standard deviation
ANOVA: Analysis of variance
AUC: Area under the ROC curve
AGE: Agarose gel electrophoresis
CV: Coefficient of variation
CI: Confidence interval
GO: Gene Ontology
KEGG: Kyoto Encyclopedia of Genes and Genomes.

## Supplementary Materials

Supplementary Materials Additional file 1. Table S1: the intra-assay CV and the interassay CV of tRF-17-WS7K092. Table S2: the diagnostic performance of tRF-17-WS7K092, CEA, CA199, and CA724 in differentiating GC patients from gastritis patients. Figure S1: tRF-17-WS7K092 is a kind of 3′-tRF. (A) UCSC Genome Browser database showed that tRF-17-WS7K092 was located at chr17 (q21.32), with 47,269,890-47,269,961. (B) Basic information about tRF-17-WS7K092 in MINTbase. (C) The cleavage site was on the T-loop of mature tRNA. (D) AGE showed a single electrophoretic band of about 80 bp for the qRT-PCR product. (E) Sanger sequencing verified the qRT-PCR product contained the complete sequence of tRF-17-WS7K092. Figure S2: comprehensive evaluation of the detection method of tRF-17-WS7K092. (A, B) The detection method of tRF-17-WS7K092 was not easily affected by these factors. (C, D) The standard curves in a tenfold serial dilution to show the linearity of serum tRF-17-WS7K092 and U6. (E, F) The amplification plot and melting plot of tRF-17-WS7K092. ^ns^*P* > 0.05.
