# Supplementary material for: Comprehensive Evaluation of Serum tRF-17-WS7K092 as a Promising Biomarker for the Diagnosis of Gastric Cancer
Source: J Oncol. 2022 Sep 19;2022:8438726. doi: 10.1155/2022/8438726 (PMC9553536; doi:10.1155/2022/8438726)
Supplement: Supplementary Materials — Additional file 1. Table S1: the intra-assay CV and the interassay CV of tRF-17-WS7K092. Table S2: the diagnostic performance of tRF-17-WS7K092, CEA, CA199, and CA724 in differentiating GC patients from gastritis patients. Figure S1: tRF-17-WS7K092 is a kind of 3′-tRF. (A) UCSC Genome Browser database showed that tRF-17-WS7K092 was located at chr17 (q21.32), with 47,269,890-47,269,961. (B) Basic information about tRF-17-WS7K092 in MINTbase. (C) The cleavage site was on the T-loop of mature tRNA. (D) AGE showed a single electrophoretic band of about 80 bp for the qRT-PCR product. (E) Sanger sequencing verified the qRT-PCR product contained the complete sequence of tRF-17-WS7K092. Figure S2: comprehensive evaluation of the detection method of tRF-17-WS7K092. (A, B) The detection method of tRF-17-WS7K092 was not easily affected by these factors. (C, D) The standard curves in a tenfold serial dilution to show the linearity of serum tRF-17-WS7K092 and U6. (E, F) The amplification plot and melting plot of tRF-17-WS7K092. nsP > 0.05. [file 8438726.f1.zip › Revised Additional file 1.docx]

**Table S1** The Intra-assay CV and the Inter-assay CV of tRF-17-WS7K092.

|  | tRF-17-WS7K092 | U6 |
| --- | --- | --- |
| **Intra-assay CV, %** | 1.8 | 2.14 |
| **Inter-assay CV, %** | 2.19 | 2.58 |

CV, coefficient of variation.

**Table S2** The diagnostic performance of tRF-17-WS7K092, CEA, CA199 and CA724 in differentiating GC patients from gastritis patients.

|  | SEN | SPE | ACCU | PPV | NPV |
| --- | --- | --- | --- | --- | --- |
| tRF-17-WS7K092 | 0.79(108/136) | 0.65(26/40) | 0.76(134/176) | 0.89(108/122) | 0.48(26/54) |
| CEA | 0.60(81/136) | 0.68(27/40) | 0.61(108/176) | 0.86(81/94) | 0.33(27/82) |
| CA199 | 0.51(69/136) | 0.78(31/40) | 0.57(100/176) | 0.88(69/78) | 0.32(31/98) |
| CA724 | 0.56(76/136) | 0.65(26/40) | 0.58(102/176) | 0.84(76/90) | 0.30(26/86) |
| tRF-17-WS7K092+CEA | 0.92(125/136) | 0.45(18/40) | 0.81(143/176) | 0.85(125/147) | 0.62(18/29) |
| tRF-17-WS7K092+CA199 | 0.93(126/136) | 0.53(21/40) | 0.84(147/176) | 0.87(126/145) | 0.68(21/31) |
| tRF-17-WS7K092+CA724 | 0.92(125/136) | 0.43(17/40) | 0.81(142/176) | 0.84(125/148) | 0.61(17/28) |
| tRF-17-WS7K092+CEA+CA199 | 0.96(131/136) | 0.35(14/40) | 0.82(145/176) | 0.83(131/157) | 0.74(14/19) |
| tRF-17-WS7K092+CEA+CA724 | 0.98(133/136) | 0.28(11/40) | 0.82(144/176) | 0.82(133/162) | 0.79(11/14) |
| tRF-17-WS7K092+CEA+CA199+CA724 | 0.99(134/136) | 0.25(10/40) | 0.82(144/176) | 0.82(134/164) | 0.83(10/12) |

SEN, sensitivity; SPE, specificity; ACCU, overall accuracy; PPV, positive predictive value; NPV, negative predictive value.


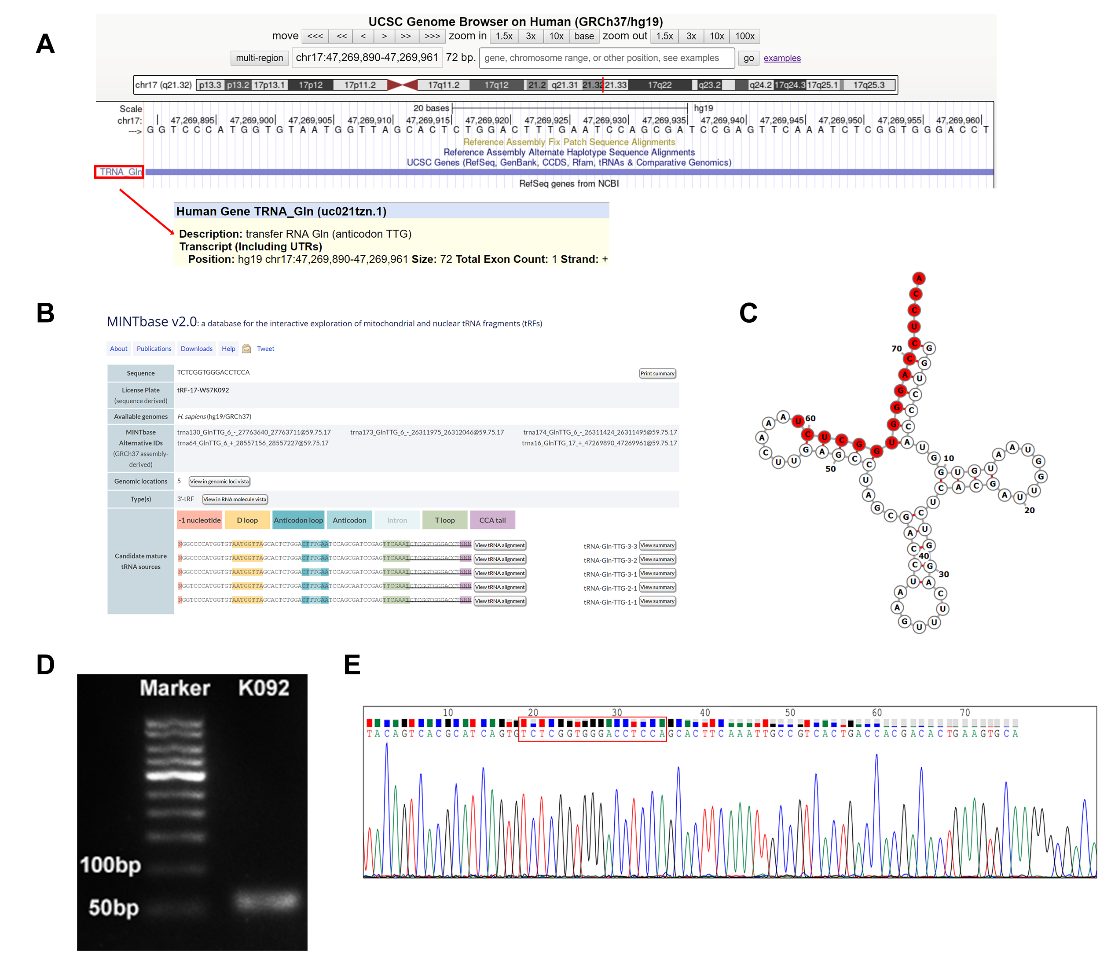


**Fig. S1.** tRF-17-WS7K092 is a kind of 3’-tRF. **A** UCSC Genome Browser database showed that tRF-17-WS7K092 was located at chr17 (q21.32), with 47,269,890-47,269,961; **B** Basic information about tRF-17-WS7K092 in MINTbase; **C** The cleavage site was on the T-loop of mature tRNA; **D** AGE showed a single electrophoretic band of about 80 bp for the qRT-PCR product; **E** Sanger sequencing verified the qRT-PCR product contain the complete sequence of tRF-17-WS7K092.


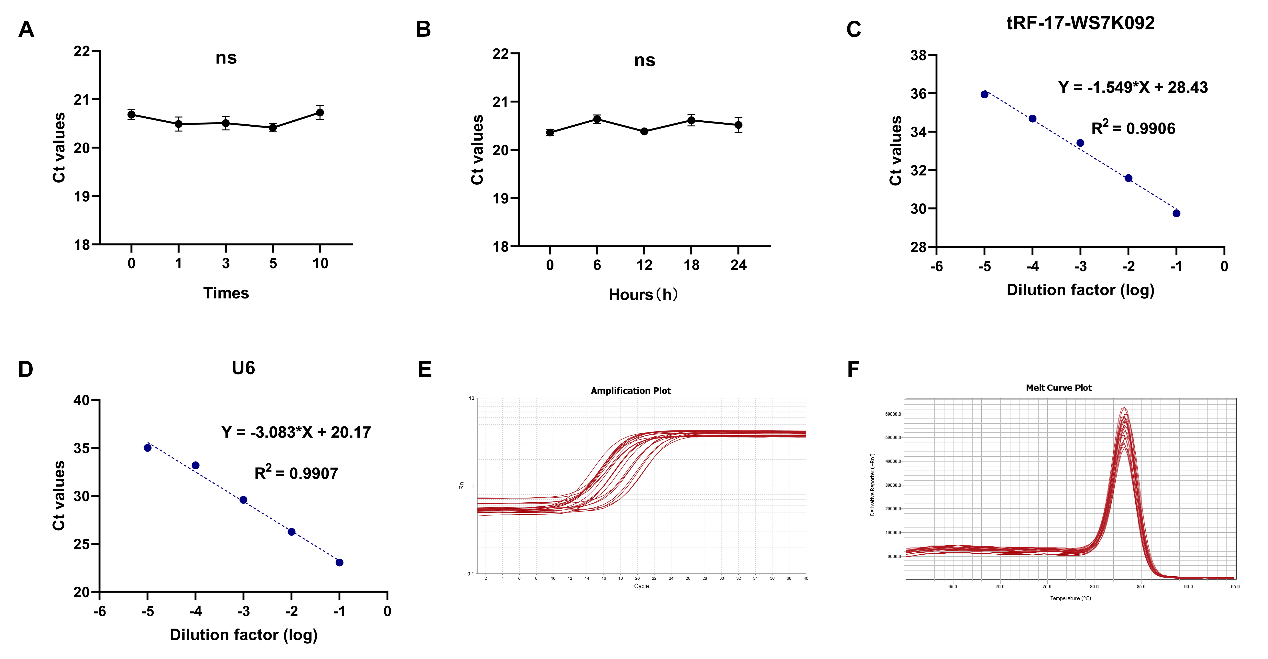


**Fig. S2.** Comprehensive evaluation of the detection method of tRF-17-WS7K092. **A, B** The detection method of tRF-17-WS7K092 was not easily affected by these factors. **C, D** The standard curves in a ten-fold serial dilution to show the linearity of serum tRF-17-WS7K092 and U6. **E, F** The amplification plot and melting plot of tRF-17-WS7K092. ^ns^P > 0.05
