# Supplementary material for: Comprehensive Evaluation of Serum tRF-17-WS7K092 as a Promising Biomarker for the Diagnosis of Gastric Cancer
Source: J Oncol. 2022 Sep 19;2022:8438726. doi: 10.1155/2022/8438726 (PMC9553536; doi:10.1155/2022/8438726)
Supplement: Supplementary Materials — Additional file 1. Table S1: the intra-assay CV and the interassay CV of tRF-17-WS7K092. Table S2: the diagnostic performance of tRF-17-WS7K092, CEA, CA199, and CA724 in differentiating GC patients from gastritis patients. Figure S1: tRF-17-WS7K092 is a kind of 3′-tRF. (A) UCSC Genome Browser database showed that tRF-17-WS7K092 was located at chr17 (q21.32), with 47,269,890-47,269,961. (B) Basic information about tRF-17-WS7K092 in MINTbase. (C) The cleavage site was on the T-loop of mature tRNA. (D) AGE showed a single electrophoretic band of about 80 bp for the qRT-PCR product. (E) Sanger sequencing verified the qRT-PCR product contained the complete sequence of tRF-17-WS7K092. Figure S2: comprehensive evaluation of the detection method of tRF-17-WS7K092. (A, B) The detection method of tRF-17-WS7K092 was not easily affected by these factors. (C, D) The standard curves in a tenfold serial dilution to show the linearity of serum tRF-17-WS7K092 and U6. (E, F) The amplification plot and melting plot of tRF-17-WS7K092. nsP > 0.05. [file 8438726.f1.zip › Revised Supplementary Table S1.docx]

**Supplementary Table S1 The Intra-assay CV and the Inter-assay CV of tRF-17-WS7K092.**

|  | tRF-17-WS7K092 | U6 |
| --- | --- | --- |
| **Intra-assay CV, %** | 1.8 | 2.14 |
| **Inter-assay CV, %** | 2.19 | 2.58 |

CV, coefficient of variation.
