# Supplementary material for: Comprehensive Evaluation of Serum tRF-17-WS7K092 as a Promising Biomarker for the Diagnosis of Gastric Cancer
Source: J Oncol. 2022 Sep 19;2022:8438726. doi: 10.1155/2022/8438726 (PMC9553536; doi:10.1155/2022/8438726)
Supplement: Supplementary Materials — Additional file 1. Table S1: the intra-assay CV and the interassay CV of tRF-17-WS7K092. Table S2: the diagnostic performance of tRF-17-WS7K092, CEA, CA199, and CA724 in differentiating GC patients from gastritis patients. Figure S1: tRF-17-WS7K092 is a kind of 3′-tRF. (A) UCSC Genome Browser database showed that tRF-17-WS7K092 was located at chr17 (q21.32), with 47,269,890-47,269,961. (B) Basic information about tRF-17-WS7K092 in MINTbase. (C) The cleavage site was on the T-loop of mature tRNA. (D) AGE showed a single electrophoretic band of about 80 bp for the qRT-PCR product. (E) Sanger sequencing verified the qRT-PCR product contained the complete sequence of tRF-17-WS7K092. Figure S2: comprehensive evaluation of the detection method of tRF-17-WS7K092. (A, B) The detection method of tRF-17-WS7K092 was not easily affected by these factors. (C, D) The standard curves in a tenfold serial dilution to show the linearity of serum tRF-17-WS7K092 and U6. (E, F) The amplification plot and melting plot of tRF-17-WS7K092. nsP > 0.05. [file 8438726.f1.zip › Revised Table 1.docx]

**Table 1 Clinicopathological analysis of tRF-17-WS7K092.**

| **Parameter** |  | **No. of patients** | **tRF-17-WS7K092(high)** | **tRF-17-WS7K092(low)** | **P-value** |
| --- | --- | --- | --- | --- | --- |
| **Sex** | male | 99 | 52 | 47 | 0.335 |
|  | female | 37 | 16 | 21 |  |
| **Age（year）** | ＜60 | 36 | 17 | 19 | 0.697 |
|  | ≥60 | 100 | 51 | 49 |  |
| **Tumor size** | ＜5 | 98 | 44 | 54 | 0.056 |
|  | ≥5 | 38 | 24 | 14 |  |
| **Differentiation grade** | Well-moderate | 63 | 27 | 36 | 0.122 |
|  | Poor-undifferentiation | 73 | 41 | 32 |  |
| **T stage** | T1-T2 | 71 | 27 | 44 | 0.004** |
|  | T3-T4 | 65 | 41 | 24 |  |
| **Lymph node status** | Positive | 85 | 52 | 33 | 0.001** |
|  | Negative | 51 | 16 | 35 |  |
| **TNM stage** | Ⅰ-Ⅱ | 76 | 28 | 48 | 0.001** |
|  | Ⅲ-Ⅳ | 60 | 40 | 20 |  |
| **Nerve/vascular invasion** | Positive | 84 | 50 | 34 | 0.005** |
|  | Negative | 52 | 18 | 34 |  |

****P<0.01**
