# Supplementary material for: Comprehensive Evaluation of Serum tRF-17-WS7K092 as a Promising Biomarker for the Diagnosis of Gastric Cancer
Source: J Oncol. 2022 Sep 19;2022:8438726. doi: 10.1155/2022/8438726 (PMC9553536; doi:10.1155/2022/8438726)
Supplement: Supplementary Materials — Additional file 1. Table S1: the intra-assay CV and the interassay CV of tRF-17-WS7K092. Table S2: the diagnostic performance of tRF-17-WS7K092, CEA, CA199, and CA724 in differentiating GC patients from gastritis patients. Figure S1: tRF-17-WS7K092 is a kind of 3′-tRF. (A) UCSC Genome Browser database showed that tRF-17-WS7K092 was located at chr17 (q21.32), with 47,269,890-47,269,961. (B) Basic information about tRF-17-WS7K092 in MINTbase. (C) The cleavage site was on the T-loop of mature tRNA. (D) AGE showed a single electrophoretic band of about 80 bp for the qRT-PCR product. (E) Sanger sequencing verified the qRT-PCR product contained the complete sequence of tRF-17-WS7K092. Figure S2: comprehensive evaluation of the detection method of tRF-17-WS7K092. (A, B) The detection method of tRF-17-WS7K092 was not easily affected by these factors. (C, D) The standard curves in a tenfold serial dilution to show the linearity of serum tRF-17-WS7K092 and U6. (E, F) The amplification plot and melting plot of tRF-17-WS7K092. nsP > 0.05. [file 8438726.f1.zip › Revised Table 2.docx]

**Table 2 The diagnostic performance of tRF-17-WS7K092, CEA, CA199 and CA724 in differentiating GC patients from healthy donors.**

|  | SEN | SPE | ACCU | PPV | NPV |
| --- | --- | --- | --- | --- | --- |
| tRF-17-WS7K092 | 0.77(105/136) | 0.84(114/136) | 0.81(219/272) | 0.83(105/127) | 0.79(114/145) |
| CEA | 0.60(81/136) | 0.71(96/136) | 0.65(177/272) | 0.67(81/121) | 0.64(96/151) |
| CA199 | 0.51(69/136) | 0.80(109/136) | 0.65(178/272) | 0.72(69/96) | 0.62(109/176) |
| CA724 | 0.56(76/136) | 0.74(101/136) | 0.65(177/272) | 0.68(76/111) | 0.63(101/161) |
| tRF-17-WS7K092+CEA | 0.90(122/136) | 0.60(81/136) | 0.75(203/272) | 0.69(122/177) | 0.85(81/95) |
| tRF-17-WS7K092+CA199 | 0.91(124/136) | 0.68(92/136) | 0.79(216/272) | 0.74(124/168) | 0.88(92/104) |
| tRF-17-WS7K092+CA724 | 0.90(123/136) | 0.63(86/136) | 0.77(209/272) | 0.71(123/173) | 0.87(86/99) |
| tRF-17-WS7K092+CEA+CA199 | 0.95(129/136) | 0.49(67/136) | 0.72(196/272) | 0.65(129/198) | 0.91(67/74) |
| tRF-17-WS7K092+CEA+CA724 | 0.96(131/136) | 0.44(60/136) | 0.70(191/272) | 0.63(131/207) | 0.92(60/65) |
| tRF-17-WS7K092+CEA+CA199+CA724 | 0.98(133/136) | 0.38(51/136) | 0.68(184/272) | 0.61(133/218) | 0.94(51/54) |

SEN, sensitivity; SPE, specificity; ACCU, overall accuracy; PPV, positive predictive value; NPV, negative predictive value.
